# Cediranib or placebo in combination with cisplatin and gemcitabine chemotherapy for patients with advanced biliary tract cancer (ABC-03): a randomised phase 2 trial

**DOI:** 10.1016/S1470-2045(15)00139-4

**Published:** 2015-08

**Authors:** Juan W Valle, Harpreet Wasan, Andre Lopes, Alison C Backen, Daniel H Palmer, Karen Morris, Marian Duggan, David Cunningham, D Alan Anthoney, Pippa Corrie, Srinivasan Madhusudan, Anthony Maraveyas, Paul J Ross, Justin S Waters, Will P Steward, Charlotte Rees, Sandy Beare, Caroline Dive, John A Bridgewater

**Affiliations:** a Institute of Cancer Studies, University of Manchester, Manchester, UK; b The Christie NHS Foundation Trust, Manchester, UK; c Imperial College Healthcare NHS Trust, London, UK; d Cancer Research UK and UCL Cancer Clinical Trials Centre, London, UK; e University of Liverpool and Clatterbridge Cancer Centre, Liverpool, UK; f The Royal Marsden NHS Foundation Trust, London, UK; g St James University Hospital, The Leeds Teaching Hospital Trust, Beckett Street, Leeds, UK; h Cambridge University Hospitals NHS Foundation Trust, Cambridge, UK; i Nottingham City Hospital, Nottingham University Hospitals NHS Trust, Nottingham, UK; j Hull York Medical School, Castle Hill Hospital, Hull, UK; k Guy's Hospital, Guy's and St Thomas' NHS Foundation Trust, London, UK; l Kent Oncology Centre, Maidstone Hospital, Kent, UK; m Leicester Royal Infirmary, University Hospitals of Leicester NHS Trust, Leicester, UK; n Southampton University Hospitals NHS Foundation Trust, Southampton, UK; o Cancer Research UK Manchester Institute, University of Manchester, Manchester, UK; p UCL Cancer Institute, London, UK

## Abstract

**Background:**

Cisplatin and gemcitabine is the standard first-line chemotherapy regimen for patients with advanced biliary tract cancer; expression of VEGF and its receptors is associated with adverse outcomes. We aimed to assess the effect of the addition of cediranib (an oral inhibitor of VEGF receptor 1, 2, and 3) to cisplatin and gemcitabine on progression-free survival.

**Methods:**

In this multicentre, placebo-controlled, randomised phase 2 study, we recruited patients aged 18 years or older with histologically confirmed or cytologically confirmed advanced biliary tract cancer from hepatobiliary oncology referral centres in the UK. Patients were eligible if they had an ECOG performance status of 0–1 and an estimated life expectancy of longer than 3 months. Patients were given first-line cisplatin and gemcitabine chemotherapy (25 mg/m^2^ cisplatin and 1000 mg/m^2^ gemcitabine [on days 1 and 8 every 21 days, for up to eight cycles]) with either 20 mg oral cediranib or placebo once a day until disease progression. We randomly assigned patients (1:1) with a minimisation algorithm, incorporating the stratification factors: extent of disease, primary disease site, previous treatment, ECOG performance status, and centre. The primary endpoint was progression-free survival in the intention-to-treat population. This study is registered with ClinicalTrials.gov, number NCT00939848, and was closed on Sept 30, 2014; results of the final analysis for the primary endpoint are presented.

**Findings:**

Between April 5, 2011, and Sept 28, 2012, we enrolled 124 patients (62 in each group). With a median follow-up of 12·2 months (IQR 7·3–18·5), median progression-free survival was 8·0 months (95% CI 6·5–9·3) in the cediranib group and 7·4 months (5·7–8·5) in the placebo group (HR 0·93, 80% CI 0·74–1·19, 95% CI 0·65–1·35; p=0·72). Patients who received cediranib had more grade 3–4 toxic effects than did patients who received placebo: hypertension (23 [37%] *vs* 13 [21%]; p=0·05), diarrhoea (eight [13%] *vs* two [3%]; p=0·05); platelet count decreased (ten [16%] *vs* four [6%]; p=0·09), white blood cell decreased (15 [24%] *vs* seven [11%]; p=0·06) and fatigue (16 [24%] *vs* seven [11%]; p=0·04).

**Interpretation:**

Cediranib did not improve the progression-free survival of patients with advanced biliary tract cancer in combination with cisplatin and gemcitabine, which remains the standard of care. Although patients in the cediranib group had more adverse events, we recorded no unexpected toxic effects. The role of VEGF inhibition in addition to chemotherapy for patients with advanced biliary tract cancer remains investigational.

**Funding:**

Cancer Research UK and AstraZeneca Pharmaceuticals.

## Introduction

Inoperable or metastatic cholangiocarcinomas, gallbladder, and ampullary cancers are collectively termed advanced biliary tract cancers. Although these diseases are deemed to be low incidence in most high-income countries (<1% of all adult cancers, with roughly 1500 cases per year in the UK^1^ and 9000 cases per year in the USA^2^), global hotspots exist with much higher incidence. Importantly, both incidence and mortality are increasing largely due to a global rise in intrahepatic cholangiocarcinoma.^3^

Most patients present with locally advanced (non-resectable) or metastatic disease and, even when surgery is feasible, recurrence is frequent. The median survival with best supportive care in randomised studies is between 2·5 and 4·5 months.^4^, ^5^ The National Cancer Research Institute (UK) Advanced Biliary tract Cancer (ABC)-02 randomised phase 3 study,^6^ established cisplatin and gemcitabine as the reference first-line chemotherapy regimen for advanced biliary tract cancer with a median overall survival of 11·7 months, a finding that was replicated in a randomised phase 2 Japanese study.^7^ However, with only half of patients with advanced disease surviving a year, improved treatment options are needed.

VEGF, one of the main growth factors regulating angiogenesis, is overexpressed in 40–75% of biliary tract cancers,^8^, ^9^, ^10^ especially at the invasive edge of the tumour.^11^ The receptors for this ligand, VEGFR1 and VEGFR2, are also overexpressed in the adjacent endothelial cells.^12^ VEGF expression is associated with the presence of metastases in intrahepatic cholangiocarcinoma;^8^ adverse prognosis in extrahepatic cholangiocarcinoma^13^ and increased microvascular density in both cholangiocarcinoma^9^ and gallbladder cancer.^10^ In turn, high microvascular density is an independent adverse prognostic factor for disease-free survival after resection of extrahepatic cholangiocarcinoma^11^ and for overall survival in lymph-node negative intrahepatic cholangiocarcinoma^14^ and gallbladder cancer.^10^ These results make angiogenesis a logical target for treatment of biliary tract cancer.

**Research in context**

**Evidence before this study**

Chemotherapy with cisplatin and gemcitabine is the reference chemotherapy regimen for patients with advanced biliary tract cancer based on the largest randomised trial in this population (ABC-02). Up to now, no randomised trial has shown superiority of any systemic therapy over this combination. Targeting angiogenesis, one of the hallmarks of cancer and a known predictor of adverse outcome in advanced biliary tract cancer, is a logical step and has proven to be effective in a number of tumour types; however, no randomised studies have been done of this approach in advanced biliary tract cancer building on the cisplatin and gemcitabine regimen.

**Added value of this study**

This study assessed the effect of adding cediranib (an oral VEGFR-1, VEGFR-2 and VEGFR-3 receptor tyrosine kinase inhibitor, with additional activity against PDGF receptors and c-KIT) to cisplatin and gemcitabine chemotherapy in a double-bind, placebo-controlled manner. The study did not meet its primary endpoint (improvement in progression-free survival); however, we recorded signals that would support further anti-angiogenesis approaches. Additionally, we showed that elevated baseline levels of the tumour markers CEA and CA125 (in addition to CA19-9) and total cytokeratin 18 and VEGFR2 are prognostic in advanced biliary tract cancer. This is the first study to show that the presence of circulating tumour cells confers an adverse prognosis, and offers an opportunity to interrogate these in future studies. Finally, baseline PDGFbb concentrations might predict for cediranib activity.

**Implications of all the available evidence**

Cisplatin and gemcitabine remains the reference regimen for patients with advanced biliary tract cancer; there is no evidence that cediranib improves progression-free survival. The effect of a better-tolerated anti-angiogenic in combination with cisplatin and gemcitabine, with consideration of our exploratory findings with respect to circulating biomarkers, warrants further investigation.

Cediranib is an oral VEGFR1, VEGFR2, and VEGFR3 tyrosine kinase inhibitor, with additional activity against PDGF receptors and c-KIT.^15^ Although the maximum tolerated dose of cediranib was not reached in a phase 1 study^16^ in patients with lung cancer, the recommended dose was 30 mg once a day in combination with cisplatin and gemcitabine (using a different schedule to that of the ABC-02 study, namely 1250 mg/m^2^ gemcitabine on days 1 and 8 and 80 mg/m^2^ cisplatin on day 1 every 3 weeks). However, based on the totality of the available safety, tolerability, efficacy, pharmacokinetic, and pharmacodynamic data at the time of study setup, the manufacturer's recommended dose of 20 mg cediranib once a day for combination with chemotherapy regimens was selected for this trial.

In this trial, ABC-03, we aimed to assess the effect of the addition of cediranib to standard cisplatin and gemcitabine chemotherapy on progression-free survival in patients with advanced biliary tract cancer.

## Methods

### Study design and participants

In this multicentre, randomised phase 2, double-blind, placebo-controlled, investigator-initiated study, we recruited patients aged 18 years or older with a histopathological or cytological diagnosis of non-resectable, recurrent, or metastatic biliary tract carcinoma (intrahepatic or extrahepatic cholangiocarcinoma), gallbladder, or ampullary carcinoma from hepatobiliary oncology referral centres in the UK. Patients were eligible if they had an Eastern Cooperative Oncology Group (ECOG) performance status of 0–1 and an estimated life expectancy of longer than 3 months.

Patients were required to have adequate haematological function (haemoglobin ≥10 g/dL; white blood cell count ≥3·0 × 10^9^ cells per L; absolute neutrophil count ≥1·5 × 10^9^ cells per L and platelet count ≥100 × 10^9^ per L), hepatic function (total bilirubin ≤1·5 times the upper limit of normal except for patients with known documented cases of Gilbert's syndrome; alanine aminotransferase or aspartate aminotransferase ≤2·5 times the upper limit of normal [if liver metastases were present, alanine aminotransferase or aspartate aminotransferase had to be less than five times the upper limit of normal] and alkaline phosphatase less than or equal to five times the upper limit of normal), and renal function (serum urea <1·5 times the upper limit of normal, serum creatinine <1·5 times the upper limit of normal, and calculated glomerular filtration rate ≥45 mL/min using a validated creatinine clearance calculation such as Cockroft-Gault or Wright formula [if the calculated glomerular filtration rate was below 45 mL/min, an isotopic method was done to confirm the glomerular filtration rate was ≥45 mL/min]). Additionally, patients could not have evidence of active uncontrolled infection (patients on long-term antibiotics were eligible provided signs of active infection had resolved) and women of childbearing potential were required to have a negative pregnancy test before study entry and to be using two methods of adequate contraception, which was to be continued for 3 months after completion of treatment.

The following previous treatment was allowed (provided there had been a full recovery): a non-curative operation (ie, R2 resection [with macroscopic residual disease] or palliative bypass surgery only); curative surgery with evidence of non-resectable disease relapse requiring systemic chemotherapy; radiotherapy (with or without radio-sensitising low-dose chemotherapy) for localised disease provided there had been clear evidence of disease progression before inclusion in this study; adjuvant chemotherapy provided neither gemcitabine nor cisplatin were used and the treatment was completed more than 6 months before trial entry; and photodynamic treatment provided that there was clear evidence of disease progression at the local site or at a new metastatic site. Previous systemic chemotherapy for locally advanced or metastatic disease was not allowed.

Patients were excluded from the study in the event of incomplete recovery (common terminology criteria for adverse events [CTCAE] grade >1) from previous anticancer treatment; unresolved biliary tree obstruction; continuing severe or uncontrolled systemic diseases which, in the view of the investigator, made it undesirable for the patient to participate in the trial (eg, unstable or uncompensated respiratory, cardiac, hepatic, or renal disease); or untreated unstable brain or meningeal metastases. Pregnant or breastfeeding women; patients with a history of cancer likely to interfere with the response assessment (exceptions include in-situ carcinoma of the cervix treated by cone-biopsy or resection, non-metastatic basal or squamous cell carcinomas of the skin, any early stage [stage I] cancer adequately resected for cure >5 years previously); and patients in receipt of treatment with an investigational drug within 30 days before randomisation were excluded.

Cediranib-directed exclusions included substantial haemorrhage (>30 mL bleeding or episode in previous 3 months) or haemoptysis (>5 mL fresh blood) within 4 weeks of randomisation; poorly controlled hypertension with resting blood pressure higher than 150/100 mm Hg in the presence or absence of a stable regimen of antihypertensive treatment, or patients requiring maximum doses of calcium channel blockers to stabilise blood pressure; proteinuria of greater than +1 on two consecutive dipsticks taken no less than 1 week apart, unless urinary protein less than 1·5 g in 24 h or protein to creatinine ratio less than 1·5; a mean QTc with Bazett's correction longer than 480 ms in screening ECG or history of familial long QT syndrome; recent major thoracic or abdominal surgery (<14 days) before randomisation, or an incompletely healed surgical incision; known hypersensitivity to cediranib or any of its excipients; or a history of gastrointestinal impairment that would substantially affect the absorption of cediranib or placebo. Because of the intensity of laboratory processing of blood products, we also excluded patients with known risk of transmitting HIV, hepatitis B or C via infected blood.

The trial was done according to the principles of the International Conference on Harmonisation of Good Clinical Practice. It was coordinated by the Cancer Research UK and University College London (UCL) Cancer Trials Centre and sponsored by UCL. All patients provided written informed consent before randomisation.

### Randomisation and masking

Patients were randomly assigned (1:1) to receive either cediranib or matching placebo in addition to cisplatin and gemcitabine chemotherapy. Treatment was assigned by computer (undertaken centrally at UCL Cancer Trials Centre, UK) with a minimisation algorithm incorporating the stratification factors: extent of disease (locally advanced *vs* metastatic), primary disease site (gallbladder *vs* bile duct *vs* ampulla), previous treatment (none *vs* adjuvant chemotherapy *vs* other), ECOG performance status (0 *vs* 1) and randomising site, and including a random element.

Supply and allocation of oral drug packs was managed by an interactive web-based response system (Cenduit, Stirling, UK). The allocated oral treatment was masked to the respective patients, treating clinician, local site staff, and UCL Cancer Trials Centre staff until analysis. An emergency unmasking procedure was established at each study site for patient safety.

### Procedures

Once deemed eligible at study screening, all patients received 25 mg/m^2^ cisplatin and 1000 mg/m^2^ gemcitabine chemotherapy, each on days 1 and 8 of a 21 day cycle, continued to eight cycles in the absence of disease progression. Additionally, patients received either 20 mg oral cediranib once a day or placebo throughout and beyond the completion of chemotherapy in the absence of disease progression. Treatment was discontinued in the event of disease progression, unacceptable adverse events, intercurrent illness preventing further treatment, a patient's decision to withdraw consent, or any alteration in the patient's condition justifying discontinuation in the site investigator's opinion. In these cases, patients remained within the trial for the purposes of follow-up and data analysis according to the treatment group to which they were allocated.

Disease status was assessed by CT scans (and MRI scans, if appropriate) at baseline, and every 3 months until disease progression (including patients who discontinued treatment for any reason other than disease progression). Response was assessed by local investigators according to RECIST (version 1.1; no central review was done); with reporting radiologists masked to the patients' treatment. Additionally, the tumour markers CA19-9, CEA, and CA125 were assessed at baseline, on day 1 of every cycle of chemotherapy, monthly when on cediranib or placebo, and then at end of treatment.

Adverse events were graded according to CTCAE (version 4.03). To proceed with administration of full dose gemcitabine and cisplatin on days 1 and 8 of each cycle the following were required: white blood cell count of 2 × 10^9^ cells per L or higher, absolute neutrophil count of 1 × 10^9^ cells per L or higher, platelets 100 × 10^9^ per L or higher, and glomerular filtration rate of 45 mL/min or higher. If gemcitabine was deferred, the cisplatin was also deferred. Day 8 treatment was deferred for toxic effect by 1 week only. If a second deferral was needed, the treatment was omitted and the patient moved on to the next treatment cycle.

Only one dose reduction for cediranib, from 20 mg once a day to 15 mg once a day (or placebo equivalent, maintaining treatment masking), was allowed. Protocol-specific guidance was provided for adverse events of interest, particularly diarrhoea, hypertension, fatigue, proteinuria, hypothyroidism, and reversible posterior leukoencephalopathy syndrome. Dose interruptions were used as the first approach to manage adverse events related to cediranib; for adverse events of grade 3 or more, dosing with cediranib or placebo was interrupted. For patients with several low-grade adverse events (eg, diarrhoea, weight loss, dehydration, and fatigue) short dose interruptions (ie, 2–5 days) of the cediranib or placebo tablets were instituted. Treatment was then restarted on resolution of the adverse event at the same dose. An interruption of up to 14 days for management of cediranib or placebo adverse events was allowed. If the adverse event promptly resolved with supportive care, cediranib or placebo restarted at the same dose along with appropriate continuing supportive care. If the adverse event was deemed related to cediranib and did not resolve to grade 1 or lower with maximum supportive care and following a dose delay of up to 14 days, cediranib or placebo was restarted at 15 mg once a day. If the adverse event failed to resolve, and it was regarded as medically appropriate, cediranib or placebo was permanently discontinued; if cediranib or placebo could not be restarted at either full or reduced dose after 14 days, it was permanently discontinued. Where possible, cediranib or placebo was suspended for 2 weeks to allow clearance before any major surgery, if required.

Blood was collected from patients for exploratory biomarker studies and processed into EDTA plasma at up to 11 timepoints; a first pretreatment baseline sample, a second pretreatment baseline sample on day 1 of the first cycle then on the first day of cycles 2–8, at the end of chemotherapy, and 1 month after the end of chemotherapy. We measured circulating markers of angiogenesis (VEGFA, VEGFC, VEGFR1, VEGFR2, Ang1, Ang2, FGFb, HGF, PDGFbb, KGF, PlGF, Tie2, and SDF1b) and inflammation (interleukin 6 and interleukin 8) with a validated multiplex ELISA platform (Aushon BioSystems, Billerica, MA, USA), according to Good Clinical Practice (GCP) standards at the Cancer Research UK Manchester Institute (Manchester, UK).^17^ Concentrations of circulating total CK18, released from epithelial cells during death (apoptosis and necrosis), were measured with an M65 ELISA (Peviva, Nacka, Sweden), also previously validated and implemented to GCP as described.^18^

Whole blood (10 mL) was collected in CellSave tubes at up to four timepoints (pretreatment baseline sample, on day 1 of cycles two and five, and 1 month after the end of chemotherapy) for enumeration of circulating tumour cells with the CellSearch platform (Janssen Diagnostics, South Raritan, NJ, USA) within 4 days of blood draw.^19^ Briefly, after immunomagnetic capture of EpCAM-positive cells, immunophenotyping using cytokeratin (CK), DAPI, and CD45 allowed classification of circulating tumour cells as EpCAM+, CK+, DAPI+, and CD45–.

Only results of the baseline circulating biomarker values are presented in this report; analysis of cell-free DNA, detailed biostatistics of longitudinal samples and exploratory analysis by subgroup (in view of the biological heterogeneity of biliary tract cancers) is in progress and will be published separately.

### Outcomes

The primary endpoint was progression-free survival; secondary endpoints were overall survival, response by RECIST (version 1.1), assessment of adverse events, and quality of life. Samples were also taken for exploratory biomarker analysis (including archival tumour tissue, and blood samples for circulating tumour cells and a panel of angiogenesis ELISAs).

### Statistical analysis

The trial was designed to directly compare progression-free survival between the two treatment groups, with 80% power and a two-sided alpha of 0·2, to detect a progression-free survival hazard ratio (HR) of 0·64. This required a sample size of 68 per group. The main analysis involved estimating the HR, 80% CI, and p value. If the HR was less than 1 and the p value was less than 0·2, the study would be deemed to have provided sufficient evidence to do a larger trial, after considering toxic effects, with a high threshold of activity. This was based on seeing an improvement in the median progression-free survival from 8 months to 12·5 months, with recruitment lasting for 18 months and patients followed up for 12 months beyond last recruitment; requiring 92 progression-free survival events. No formal interim analysis was planned or done for this trial. During the conduct of the study, the end of recruitment became fixed by date (Sept 30, 2012) rather than by accrual following the decision by the manufacturer to cease development of cediranib; consequently, the power of the study was reduced to 75% and the target sample size adjusted accordingly to include at least 116 patients.

We plotted Kaplan-Meier curves for progression-free survival and overall survival; treatment effect HR (with 80% and 95% CIs and p values) were obtained from Cox proportional hazards regression models. An HR lower than 1 favoured the cediranib group. The proportionality assumption of the Cox model was tested with Schoenfeld residuals. We calculated adjusted and unadjusted HR and reported unadjusted HR. The principal analysis included all patients by allocated treatment group on an intention-to-treat basis. Survival endpoints were measured from date of randomisation to date of event. For progression-free survival, symptomatic deterioration was accepted as disease progression (patients with a global deterioration of health status requiring discontinuation of treatment without objective evidence of disease progression at that time were classified as having symptomatic deterioration).

Best overall response was summarised as frequencies, and the difference of proportions test (with 95% CI and p value) was used to compare responses and disease control between the two groups. Only patients with measurable disease at baseline were included in this analysis. Duration of response was measured from the date the best overall response was documented to the date of progression-free survival event.

All treatment-emergent adverse events reported are presented and summarised as frequencies; we used Pearson's χ^2^ test of association to formally compare grade 3–4 adverse events occurring in at least ten patients. We used the Kaplan-Meier method and Cox regression model to compare the median time on each treatment.

The associations of biomarkers (ELISA, total CK18 [M65], circulating tumour cell count, CEA, CA19-9, and CA125) with overall survival and progression-free survival were assessed with Cox regression models. The biomarkers were included in the Cox models as continuous and categorical variables. All overall survival and progression-free survival models were adjusted for treatment group and baseline characteristics (age, sex, ECOG performance status, primary site, stage, and previous treatment). Overall survival and progression-free survival Cox models were used to test for interactions between treatment and each biomarker (handled as continuous and binary variables). Treatment effect on overall survival and progression-free survival were assessed for lower and higher values of each baseline biomarker. Kaplan-Meier curves were used to graphically represent the findings. All Cox models were assessed for the proportionality of hazards assumption. No correction was made for multiple testing.

All analyses were done in Stata (version 12). This study is registered with ClinicalTrials.gov, number NCT00939848.

### Role of the funding source

The academic chief investigator (JWV), in conjunction with UCL, was responsible for the study design and conduct including the collection, management, analysis, and interpretation of the data; along with the preparation, review, and approval of the presentation and publication of the study. Study conduct was overseen by an independent trial steering committee. The trial management group had access to all the raw data and take full responsibility for the integrity of the data and accuracy of the data analysis. The chief investigator had full access to all the data in the study and had final responsibility for the decision to submit for publication.

## Results

**Figure 1:**
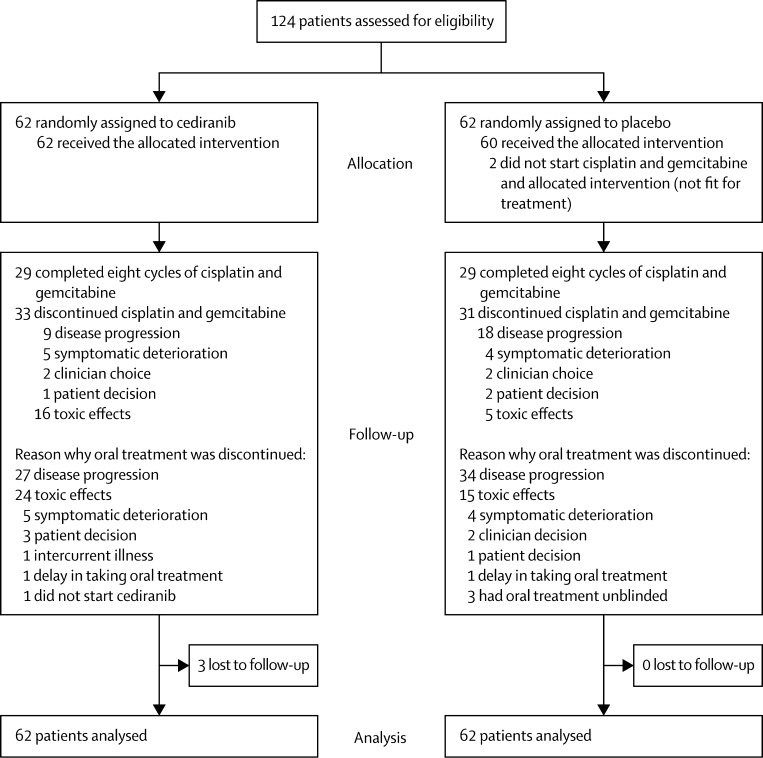
Trial profile

Between April 5, 2011, and Sept 28, 2012, 124 patients were recruited from 14 UK sites (tertiary hepatobiliary oncology referral centres; appendix). Figure 1 shows the trial profile. Table 1 shows the patient characteristics at baseline. The median patient age was 65·1 years, 50% of patients were men, and most patients (84%) had metastatic disease. Most patients had primary tumours arising from the bile ducts (62%) followed by gallbladder cancer (31%) and ampullary tumours (6%); there was a slight preponderance of patients with performance status of 1 (56%) compared with a performance status of zero (44%). At the time of analysis (April 28, 2014), the estimated median follow-up in all patients using censored deaths method was 12·2 months (IQR 7·3–18.5); 116 (94%) patients had experienced disease progression and 100 (81%) patients had died.

Table 2 summarises the incidence of adverse events by grade 1–2 and grade 3–4. We noted no significant difference between treatment groups with respect to grade 3–4 haematological toxic effects, although patients receiving cediranib had more frequent grade 3–4 decreased platelet counts (table 2). We recorded a significantly higher incidence of grade 3–4 hypertension, diarrhoea, and fatigue in patients given cediranib than in those given placebo (table 2). 88 patient deaths were related to biliary tract cancer (43 [86%] in the cediranib group and 45 [90%] in the placebo group). In the cediranib group, there was one death attributed to treatment (myocardial infarction) and one death related to both cancer and treatment (gastric haemorrhage). In the placebo group, there were three deaths attributed to treatment (one due to peripheral ischaemia and stroke; one due to cardiac arrest; and one due to pulmonary embolism, superior vena cava obstruction, and metastatic gallbladder cancer).

There was no significant difference in the median duration of treatment (ie, time on treatment) with cediranib compared with placebo (4·6 months [95% CI 2·8–8·0] with cediranib *vs* 5·5 months [2·8–7·8] with placebo, HR 1·00 [95% CI 0·70–1·44]; p=0·98). However, more patients in the cediranib group discontinued oral treatment because of toxic effects (24 in the cediranib group *vs* 15 in the placebo group; figure 1). Additionally, 14 (23%) of 62 patients had at least one dose reduction and 60 (97%) had at least one treatment suspension in a specific cycle or month in the cediranib group compared with four (7%) of 60 and 52 (87%) of 60 patients in the placebo group, respectively. Allocation of patients to cediranib did not affect the delivered dose intensity of either cisplatin or gemcitabine (data not shown).

**Figure 2:**
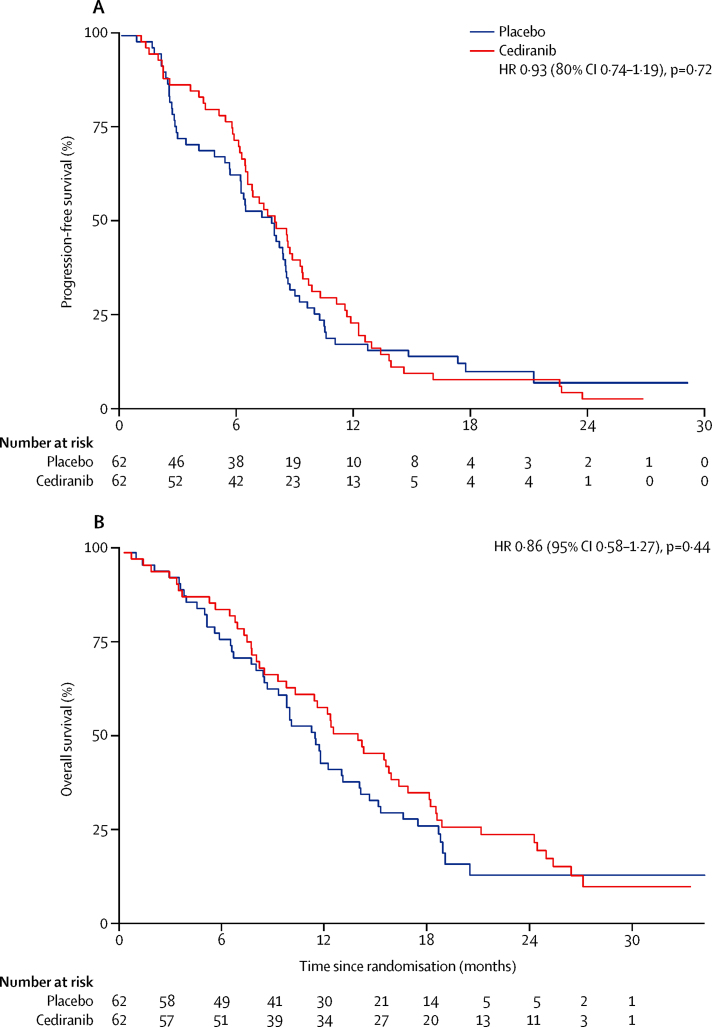
Kaplan-Meier curves for progression-free survival (A), and overall survival (B) by treatment

At the time of data cutoff, 59 progression-free survival events had occurred in the cediranib group as had 57 in the placebo group. We noted no significant difference in median progression-free survival, the primary endpoint of the study, between patients given cediranib versus placebo (8·0 months [95% CI 6·5–9·3] with cediranib *vs* 7·4 months [5·7–8·5] with placebo, HR 0·93 [80% CI 0·74–1·19, 95% CI 0·65–1·35]; p=0·72). The proportionality assumption of the Cox model used to establish the HR was tested and found to hold (p=0·09). 6-month progression-free survival was 70·5% (95% CI 57·4–80·3) in patients receiving cediranib and 61·3% (48·0–72·1) in patients receiving placebo; 12-month progression-free survival was 21·8% (95% CI 12·4–32·9) in the cediranib group and 16·1% (8·3–26·3) in the placebo group (figure 2A). Stratified analyses based on sex, age, ECOG performance status, primary tumour site, disease status, histological grade, previous treatment, and CA19-9 did not identify any outliers (appendix).

Most patients (113 [91%]; 59 in the cediranib group and 54 in the placebo group) had measurable disease at baseline. Radiological assessment by Response Evaluation Criteria In Solid Tumours (RECIST [version 1.1]) showed a greater number of responses in the cediranib group compared with the placebo group (26 [44%], including two [3%] complete responses and 24 [41%] partial responses in the cediranib group *vs* ten [19%] in the placebo group, all of which were partial responses; p=0·0036). Stable disease was noted in 20 (34%) patients in the cediranib group and 25 (46%) in the placebo group. As a result, disease control was achieved by 46 (78%) patients in the cediranib group and by 35 (65%) in the placebo group (p=0·12). The median duration of response was 5·1 months (range 2·2–24) in the cediranib group (26 events in 26 patients) and 5·8 months (0·5–22·4) in the placebo group (seven events in ten patients). Six (10%) patients in the cediranib group and 15 (28%) in the placebo group had progressive disease (including symptomatic progressions [ie, not objectively obsessed] in three in the cediranib group and four in the placebo group). Seven (12%) patients in the cediranib group and four (7%) in the placebo group had an unknown best overall response.

At data cutoff, 50 (81%) patients in the cediranib group and 50 (81%) in the placebo group had died. Median overall survival was 14·1 months (95% CI 10·2–16·4) in patients given cediranib and 11·9 months (9·2–14·3) in patients given placebo (HR 0·86, 95% CI 0·58–1·27; p=0·44). 6-month overall survival was 85·3% (95% CI 73·6–92·1) in the cediranib group and 79·0% (66·6–87·2) in the placebo group; at 12 months, 58·1% (95% CI 44·5–69·4) of patients receiving cediranib were alive compared with 48·4% (35·5–60·1) of patients in the placebo group (figure 2B). We recorded no difference in median overall survival post-progression on first-line treatment received in this study (cediranib 4·5 months [95% CI 2·8–6·4], placebo 4·3 months [3·3–6·1], HR 0·9 [0·59–1·37]; p=0·62).

Baseline CA19-9 was raised in 71 (68%) of 105 patients; an additional 23 (22%) of patients with normal CA19-9 concentrations had an increased baseline CEA or CA125; and 11 (10%) patients had normal levels of all three tumour markers (appendix). Cox models for the association between baseline tumour markers and overall survival (treatment-adjusted HRs) showed that increased baseline CA19-9 concentration was associated with an increased risk of death (table 3). This was also the case with raised CEA and CA125 (table 3). Circulating total CK18 concentrations were also prognostic, with raised baseline concentrations associated with a shorter overall survival (table 3).

**Figure 3:**
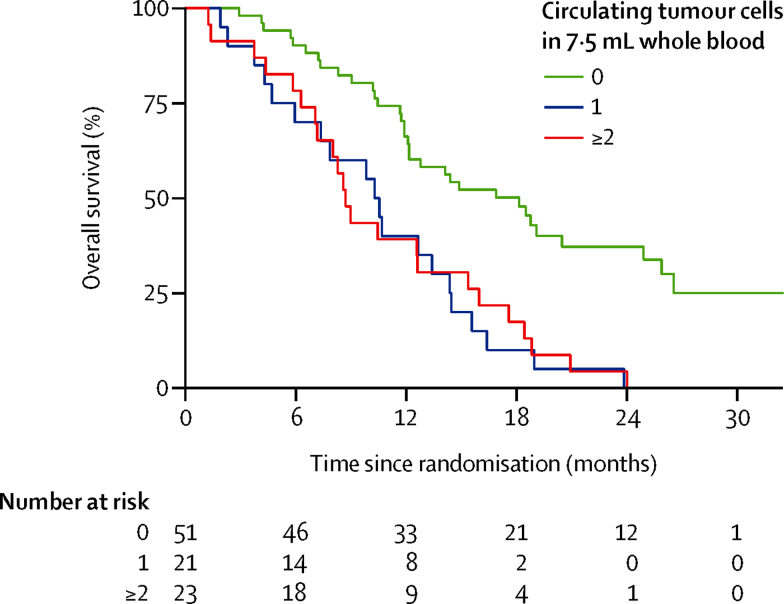
Kaplan-Meier curves for overall survival, by number of circulating tumour cells in 7·5 mL whole blood, collected before treatment

Blood samples for circulating tumour cell enumeration were available from 95 patients before treatment; 51 (54%) of 95 patients had a circulating tumour cell count of no cells per 7·5 mL blood, 21 (22%) had a count of one cell per 7·5 mL blood, and 23 (24%) had a count of two cells or higher per 7·5 mL blood, with a median of 0 per 7·5 mL blood and mean of 2 per 7·5 mL blood (range 0–44 per 7·5 mL blood). We noted an association between the presence of circulating tumour cells (based on the CellSearch criteria) and increased risk of death (HR 1·05, 95% CI 1·02–1·09); p=0·001; table 3). This same pattern was shown when the circulating tumour cell variable was divided into tertiles (1 *v*s 0, HR 3·25 [95% CI 1·81–5·83]; ≥2 *vs* 0, HR 3·00 [1·73–5·22], p<0·0001; figure 3). Patients with no circulating tumour cells in their blood sample had the best overall survival (median 18·1 months [95% CI 12·1–24·9]); the presence of one (median 10·3 months [95% CI 4·7–14·4]) or two or more circulating tumour cells (median 8·7 months [95% CI 7·1–12·6]) was strongly associated with a worse overall survival.

**Figure 4:**
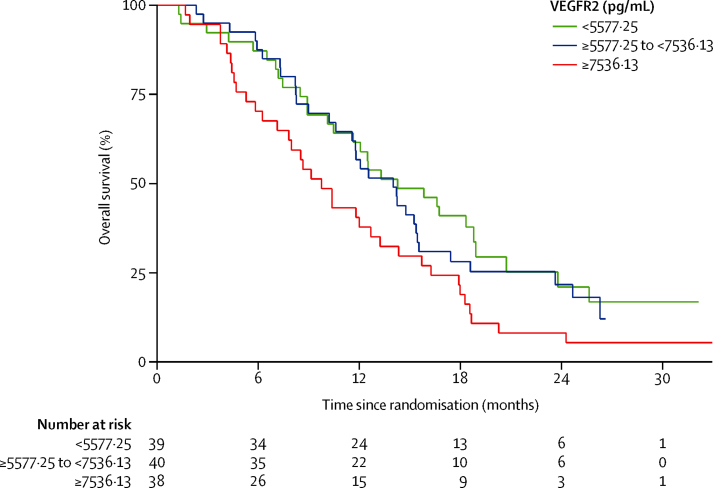
Kaplan-Meier curves for overall survival, by number of circulating VEGFR2 in the plasma, before treatment

Most of the circulating angiogenesis-related biomarkers (VEGFA, VEGFC, VEGFR1, Ang1, Ang2, FGFb, HGF, PDGFbb, KGF, PlGF, Tie2, and SDF1b) and the inflammatory biomarkers (interleukin 6 and interleukin 8) did not show prognostic significance for overall survival (data not shown). However, when adjusted for treatment and baseline characteristics, we noted that raised concentrations of baseline VEGFR2 were associated with an increased risk of death (table 3). This same pattern could be seen when we assessed VEGFR2 by tertiles (figure 4).

**Figure 5:**
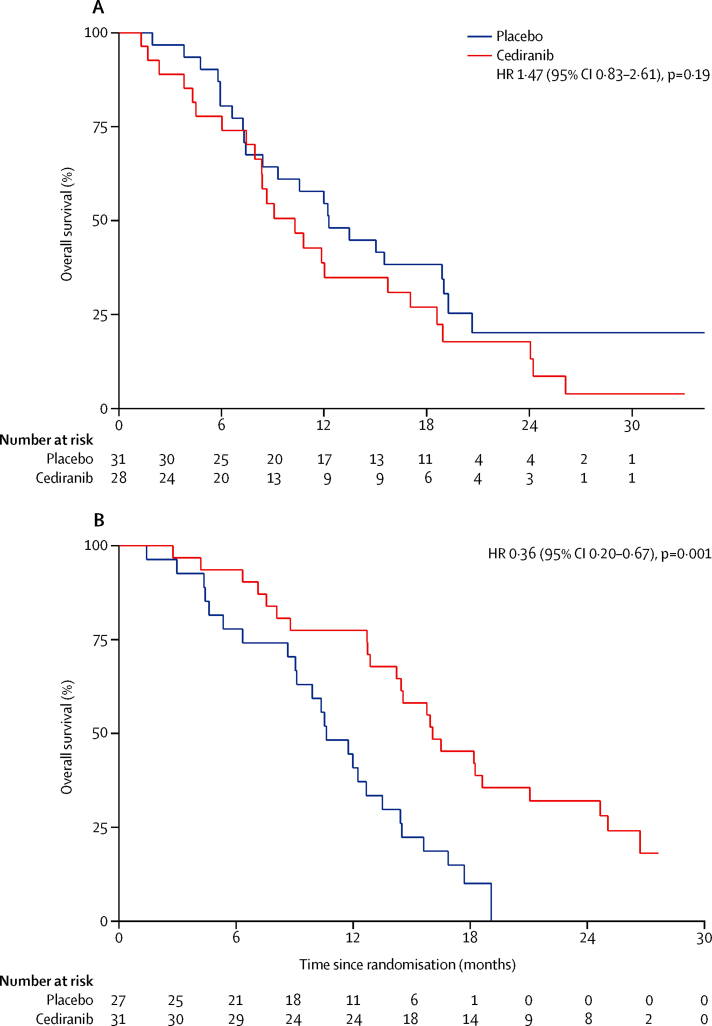
Kaplan-Meier curves for overall survival, by concentrations of circulating PDGFbb in the plasma, before treatment Plots show treatment effect by low (A) and high concentrations (B) of PDGFbb at baseline (median 162 pg/mL PDGFbb is the cutoff point), which show evidence of interaction between PDGFbb and treatment.

We recorded an interaction between baseline PDGFbb concentrations and treatment with cediranib for overall survival; with the median as the cutoff, we noted that patients with concentrations below the median did not benefit from cediranib (figure 5A) whereas patients with PDGFbb concentrations higher than the median derived benefit from cediranib in terms of overall survival (p_interaction_=0·002; figure 5B and appendix). With respect to progression-free survival, our findings suggest that the risk of having a progression-free survival event was reduced in the cediranib group compared with placebo at higher baseline levels of PDGFbb, interleukin 6, interleukin 8, PlGF, VEGFA, and Ang1 (appendix).

## Discussion

In this multicentre, double-blind placebo-controlled trial the addition of cediranib to first-line cisplatin and gemcitabine chemotherapy did not improve the progression-free survival of patients with advanced biliary tract cancer; thus, cisplatin and gemcitabine remains a standard treatment for these patients.

Based on preclinical and clinical data supporting a VEGF-targeted approach, several investigators have pursued this approach. Earlier studies have provided only limited information, largely because of their single-group phase 2 design. Sorafenib (an oral multi-tyrosine kinase inhibitor) was deemed to have low activity as monotherapy, albeit in a mainly second-line setting;^20^ bevacizumab (a monoclonal antibody against VEGF) with erlotinib (an oral EGFR inhibitor) was feasible in the first-line setting although, with a median overall survival of 9·9 months, does not seem better than upfront chemotherapy.^21^ In another study,^22^ bevacizumab was tolerable in combination with gemcitabine and oxaliplatin; however, we could not work out the incremental value of the anti-angiogenic in this single-group study. Most recently (contemporaneous to our study), sorafenib has failed to improve progression-free survival when added to gemcitabine in a randomised phase 2, placebo-controlled study of 102 patients.^23^

Our approach is the first to build on the cisplatin and gemcitabine chemotherapy regimen, now established as a reference regimen; we assessed the effect of adding cediranib, a pan-VEGF receptor tyrosine kinase inhibitor with activity against PDGF receptors and c-KIT in a placebo-controlled manner. The study did not meet its primary endpoint (to detect a progression-free survival HR of 0·64). Despite this shortcoming, some efficacy findings warrant additional attention; namely significantly greater number of overall responses and proportion of patients who achieved disease control in those receiving cediranib, mainly at the first (3 month) reassessment scan. Response is notoriously difficult to assess in patients with biliary tract cancer, although the risk of bias this could introduce was mitigated by the use of a placebo control in this study. Irrespective, patients remained on cediranib for a median of 4·6 months, stopping earlier than the median progression-free survival (8·0 months), mainly because of toxic effects; moreover, the proportion of patients stopping treatment because of toxic effects was higher in the cediranib group than the placebo group. An examination of the progression-free survival Kaplan-Meier curves suggests a separation in the period during which cediranib was tolerated, which rapidly converged after the median cessation point of cediranib. Consistent with this finding is the numerically greater number of responses in the cediranib group. These data suggest that cediranib is effective during the period of treatment but toxic effects leading to cessation prevent longer-term benefit; we postulate that a better-tolerated anti-VEGF treatment might potentially result in overall benefit in combination with cisplatin and gemcitabine.

We did not note any unexpected safety signals; the addition of cediranib to cisplatin and gemcitabine did result in a significantly greater incidence of grade 3–4 toxic effects, which were mainly expected off-target toxic effects for VEGF inhibitors (namely hypertension and diarrhoea). There were three deaths attributed to treatment in each group, with no clear additional effect from cediranib. However, these toxic effects were clearly significant in this study and need to be considered in any future angiogenesis-targeting studies.

The effect on the quality of life of patients is important in the event that the study had shown benefit from the addition of cediranib as part of the assessment of the relative merits of a potentially new agent. However, because there was no improvement in progression-free survival, quality-of-life findings from this study will be reported separately, most likely in a pooled analysis with that of other studies of advanced biliary tract cancer.

We assessed surrogates of disease volume and biology (other than radiological extent) including assessment of the tumour markers CA19-9, CEA, and CA125 and total CK18. The role of CA19-9 as a marker of volume of disease, prognostic factor, and measure of response to treatment is well documented.^24^ However, because this is linked to the Lewis blood-group antigen, not all patients express this (32% were CA19-9-negative in our series). We have been able to show that in roughly one in five cases (22%), CEA or CA125 might be used as an alternative and that each of these also has prognostic value, with increased baseline levels independently correlating with an adverse overall survival.

Cancers of epithelial origin contain large intracellular pools of cytokeratins; during necrotic or apoptotic cell death CK18 and other cytokeratins are released into the blood in either their intact or their caspase-cleaved forms where they remain stable in the circulation.^25^ CK18 is proposed as a surrogate biomarker of drug-induced epithelial cell death^26^ and thus might not only represent epithelial tumour cell death but may also reflect toxic effects in epithelial host tissues. We have shown here for the first time, to the best of our knowledge, that increased baseline CK18 levels are adversely prognostic in biliary tract cancer, consistent with our previous findings in colorectal cancer.^27^

The presence of circulating tumour cells is prognostic in a number of cancer types including prostate, breast, and colorectal cancers where CellSearch enumeration of circulating tumour cells is approved by the US Food and Drug Administration for prognosis and monitoring. We have shown that circulating tumour cells number by CellSearch is also prognostic in lung cancer,^28^, ^29^ pancreatic cancer,^30^ hepatocellular carcinoma,^31^ and melanoma.^32^ Our study is, to the best of our knowledge, the first to show the presence of circulating tumour cells in patients with biliary tract cancer and, moreover, that the presence of one or more circulating tumour cells per 7·5 mL of blood is associated with an adverse prognosis. This finding (subject to further validation) could not only inform future study design, but might also serve as a platform for longitudinal surveillance of disease behaviour both in terms of circulating tumour cell enumeration and detailed molecular analysis of individual cells.

As previously discussed, VEGF and its receptors are overexpressed in patients with biliary tract cancer; correlating with the presence of metastases and an adverse prognosis. Baseline VEGFR2 levels are associated with progression-free survival (but not overall survival) in cediranib studies in hepatocellular carcinoma,^33^ although were not prognostic (for progression-free survival or overall survival) in colorectal cancer.^34^ Assessment of a panel of angiogenesis-associated biomarkers in our study identified VEGFR2 as a novel adverse prognostic marker for overall survival in this disease group; however, despite this being a major target of cediranib with pharmacodynamic properties reported in the phase 1 study,^35^ baseline concentrations were not associated with a treatment effect (ie, were not predictive).

PDGF, a mitogen chemotactic factor promoting wound healing, neoplastic transformation, and tumour pathogenesis, is a dimeric molecule that, when active, exists either as a homodimer or a heterodimer of a and b chains.^36^ In-vitro and in-vivo studies have identified that myofibroblast-derived PDGFbb is potentially cytoprotective to cholangiocarcinoma cells by inhibition of the TRAIL (death ligand) cytotoxicity.^37^ In our study, increased baseline levels of PDGFbb, one of the targets of cediranib, was associated with a benefit from cediranib consistent with a potential role as a predictive biomarker; this finding would need to be validated in an independent cohort.

In summary, the addition of cediranib to cisplatin and gemcitabine chemotherapy did not improve progression-free survival. However, VEGF inhibition might still be an avenue worth pursuing, perhaps with an agent that has a toxic effect profile that allows more sustained dosing with cisplatin and gemcitabine than cediranib. Integration into future trial designs of the circulating biomarkers identified in our study (VEGFR2, total CK18 in addition to the tumour markers CA125, CEA, and CA19-9) will depend on validation of their utility. Finally, our identification of circulating tumour cells in patients with biliary tract cancers will allow risk stratification via circulating tumour cell enumeration and, coupled with our recently developed protocols for circulating tumour cell isolation and single cell genomic and transcriptomic profiling,^38^, ^39^ could open up new avenues for minimally invasive monitoring and delivery of precision medicine for these patients.

## Figures and Tables

**Table 1:** Baseline characteristics

			**Cediranib group (n=62)**	**Placebo group (n=62)**
Age (years)			68·0 (60·4–73·0)	64·5 (59·7–73·1)
Sex				
	Female		28 (45%)	34 (55%)
	Male		34 (55%)	28 (45%)
Primary tumour site				
	Cholangiocarcinoma		38 (61%)	39 (63%)
		Intrahepatic	14 (23%)	15 (24%)
		Extrahepatic	24 (39%)	24 (39%)
	Gallbladder		20 (32%)	19 (31%)
	Ampulla		4 (6%)	4 (6%)
Histological grade				
	Well differentiated		26 (42%)	27 (44%)
	Moderately differentiated		20 (32%)	21 (34%)
	Poorly differentiated		13 (21%)	13 (21%)
	Not specified		3 (5%)	1 (2%)
Previous treatment				
	Adjuvant chemotherapy		2 (3%)	1 (2%)
	Other*		23 (37%)	22 (35%)
	None		37 (60%)	39 (63%)
ECOG performance status				
	0		27 (44%)	28 (45%)
	1		35 (56%)	34 (55%)
Disease status				
	Locally advanced		12 (19%)	8 (13%)
	Metastatic		50 (81%)	54 (87%)
		Liver	36 (58%)	34 (55%)
		Peritoneum	19 (31%)	5 (8%)
		Omentum	5 (8%)	4 (6%)
		Lung	6 (10%)	9 (15%)
CA19-9 (IU/mL)†			298 (38–2258)	53 (10–492)

Data are median (IQR) or n (%). ECOG=Eastern Cooperative Oncology Group.

* 33 patients had surgery only (16 in the cediranib group and 17 in the placebo group) and 12 had surgery plus biliary stent insertions (seven in the cediranib group and five in the placebo group).

† CA19-9 was assessable in 59 patients in each group.

**Table 2:** Treatment-emergent adverse events arising in ≥10% patients, or of special interest, irrespective of cause

	**Cediranib group (N=62)**		**Placebo group (N=62)**		p value*
		Grade 1–2	Grade 3–4	Grade 1–2	Grade 3–4
**Haematological adverse events**					
Anaemia	51 (82%)	8 (13%)	49 (79%)	8 (13%)	0·99
Platelet count decreased	34 (55%)	10 (16%)	28 (45%)	4 (6%)	0·09
White blood cell decreased	28 (45%)	15 (24%)	32 (52%)	7 (11%)	0·06
Neutrophil count decreased	22 (35%)	26 (42%)	20 (32%)	23 (37%)	0·58
Any haematological adverse event	13 (21%)	49 (79%)	12 (19%)	49 (79%)	0·99
**Liver function adverse events**					
Increased alanine aminotransferase	43 (69%)	7 (11%)	39 (63%)	4 (6%)	0·34
Increased alkaline phosphatase	43 (69%)	4 (6%)	43 (69%)	4 (6%)	0·99
Increased aspartate aminotransferase	40 (65%)	3 (5%)	35 (56%)	2 (3%)	0·65
Increased gamma-glutamyl transpeptidase	32 (52%)	29 (47%)	23 (37%)	31 (50%)	0·72
Increased blood bilirubin	9 (15%)	3 (5%)	9 (15%)	4 (6%)	0·70
**Non-haematological adverse events**					
Constipation	42 (68%)	0 (0%)	42 (68%)	0 (0%)	..
Anorexia	40 (65%)	3 (5%)	25 (40%)	2 (3%)	..
Nausea	39 (63%)	4 (6%)	42 (68%)	1 (2%)	..
Diarrhoea	37 (60%)	8 (13%)	22 (35%)	2 (3%)	0·05
Fatigue	36 (58%)	16 (26%)	40 (65%)	7 (11%)	0·04
Lethargy	35 (56%)	8 (13%)	35 (56%)	7 (11%)	0·78
Hypoalbuminaemia	31 (50%)	0 (0%)	23 (37%)	0 (0%)	..
Abdominal pain	28 (45%)	4 (6%)	27 (44%)	5 (8%)	..
Hyponatraemia	26 (42%)	10 (16%)	28 (45%)	4 (6%)	0·09
Hypercalcaemia	25 (40%)	2 (3%)	22 (35%)	0 (0%)	..
Vomiting	24 (39%)	6 (10%)	21 (34%)	9 (15%)	0·41
Hypomagnesaemia	23 (37%)	7 (11%)	18 (29%)	3 (5%)	0·19
Mucositis oral	23 (37%)	2 (3%)	13 (21%)	0 (0%)	..
Hypophosphataemia	23 (37%)	2 (3%)	17 (27%)	2 (3%)	..
Oedema limbs	22 (35%)	0 (0%)	16 (26%)	1 (2%)	..
Pain	22 (35%)	8 (13%)	25 (40%)	4 (6%)	0·22
Dysgeusia	20 (32%)	0 (0%)	19 (31%)	0 (0%)	..
Hypokalaemia	20 (32%)	1 (2%)	18 (29%)	0 (0%)	..
Hypertension	19 (31%)	23 (37%)	17 (27%)	13 (21%)	0·05
Creatinine increased	17 (27%)	0 (0%)	10 (16%)	0 (0%)	..
Peripheral sensory neuropathy	15 (24%)	1 (2%)	16 (26%)	0 (0%)	..
Fever	15 (24%)	3 (5%)	14 (23%)	1 (2%)	..
Dyspnoea	15 (24%)	1 (2%)	16 (26%)	3 (5%)	..
Epistaxis	15 (24%)	2 (3%)	6 (10%)	0 (0%)	..
Dry mouth	14 (23%)	1 (2%)	8 (13%)	0 (0%)	..
Upper respiratory infection	14 (23%)	1 (2%)	5 (8%)	4 (6%)	..
Cough	13 (21%)	0 (0%)	8 (13%)	0 (0%)	..
Dyspepsia	13 (21%)	0 (0%)	7 (11%)	0 (0%)	..
Flu-like symptoms	12 (19%)	0 (0%)	12 (19%)	0 (0%)	..
Back pain	12 (19%)	3 (5%)	10 (16%)	1 (2%)	..
Generalised muscle weakness	12 (19%)	1 (2%)	6 (10%)	0 (0%)	..
Rash maculopapular	12 (19%)	0 (0%)	5 (8%)	1 (2%)	..
Sore throat	11 (18%)	0 (0%)	6 (10%)	0 (0%)	..
Weight loss	11 (18%)	1 (2%)	4 (6%)	0 (0%)	..
Gastro-oesophageal reflux disease	10 (16%)	0 (0%)	17 (27%)	0 (0%)	..
Insomnia	10 (16%)	1 (2%)	12 (19%)	0 (0%)	..
Alopecia	10 (16%)	0 (0%)	18 (29%)	0 (0%)	..
Urinary tract infection	9 (15%)	0 (0%)	5 (8%)	2 (3%)	..
Pain in extremity	8 (13%)	0 (0%)	5 (8%)	1 (2%)	..
Myalgia	7 (11%)	0 (0%)	11 (18%)	0 (0%)	..
Papulopustular rash	7 (11%)	0 (0%)	3 (5%)	0 (0%)	..
Flatulence	7 (11%)	0 (0%)	6 (10%)	0 (0%)	..
Dizziness	6 (10%)	0 (0%)	8 (13%)	0 (0%)	..
Mucosal infection	6 (10%)	0 (0%)	2 (3%)	0 (0%)	..
Chills	5 (8%)	0 (0%)	2 (3%)	0 (0%)	..
Biliary tract infection	3 (5%)	5 (8%)	0 (0%)	3 (5%)	..
Tinnitus	3 (5%)	1 (2%)	9 (15%)	0 (0%)	..
Hearing impaired	2 (3%)	1 (2%)	7 (11%)	0 (0%)	..
Lung infection	2 (3%)	2 (3%)	9 (15%)	2 (3%)	..
Sepsis	1 (2%)	5 (8%)	1 (2%)	2 (3%)	..
Thromboembolic event	0 (0%)	7 (11%)	1 (2%)	8 (13%)	0·78
Febrile neutropenia	0 (0%)	4 (6%)	0 (0%)	1 (2%)	..
Myocardial Infarction	0 (0%)	1 (2%)	0 (0%)	0 (0%)	..
Non-cardiac chest pain	0 (0%)	2 (3%)	6 (10%)	0 (0%)	..
Hepatobiliary disorder—biliary obstruction	0 (0%)	0 (0%)	0 (0%)	4 (6%)	..
Hepatic infection	0 (0%)	0 (0%)	0 (0%)	1 (2%)	..

Data are n (%).

^†^Five patients had a grade 5 adverse event, three in the cediranib group (one myocardial infarction, one cerebrovascular accident, one gastric haemorrhage) and two in the placebo group (one cholangitis, one peripheral ischaemia).

* Comparison of incidence of grade 3–4 events in at least ten patients (Pearson's χ^2^ test).

**Table 3:** Associations between baseline biomarkers and progression-free survival and overall survival

	**N**	**Overall survival (adjusted for treatment only)**			Overall survival (adjusted for treatment and baseline characteristics*)		**PFS (adjusted for treatment only)**		
		Deaths	HR (95% CI)	p value	HR (95% CI)	p value	PFS events	HR (95% CI)	p value
CEA (μg/L)/10	123	99	1·03 (1·01–1·04)	<0·001	1·03 (1·02–1·05)	<0·001	115	1·03 (1·01–1·04)	0·001
CA19-9 (IU/mL)/10 000	118	97	1·03 (1·01–1·05)	0·02	1·03 (1·01–1·05)	0·03	110	1·24 (1·04–1·50)	0·02
CA125 (IU/mL)/100	110	88	1·05 (1·01–1·10)	0·03	1·08 (1·03–1·13)	0·001	103	1·08 (1·03–1·13)	0·001
Total CK18 (M65; U/L)/100	119	97	1·03 (1·00–1·05)	0·03	1·04 (1·01–1·06)	0·005	112	1·02 (1·00–1·04)	0·06
CTC count (cells per 7·5 mL)	95	76	1·05 (1·02–1·09)	0·001	1·06 (1·02–1·11)	0·002	89	1·04 (1·01–1·07)	0·01
VEGFR2 (pg/mL)/1000	117	96	1·07 (0·99–1·16)	0·09	1·10 (1·01–1·20)	0·02	110	1·04 (0·96–1·12)	0·34

PFS=progression-free survival. HR=hazard ratio. CTC=circulating tumour cell. The /XX shows the per unit increase; for example, for CEA for every 10 μg/L increase, the hazard increases by 3% (HR 1·03).

* Baseline characteristics: age, sex, Eastern Cooperative Oncology Group, primary site, stage, previous treatment.
