# Correction to *Lancet Oncol* 2015; 16: 967–78

**DOI:** 10.1016/S1470-2045(15)00239-9

**Published:** 2015-09

*Valle JW, Wasan H, Lopes A, et al. Cediranib or placebo in combination with cisplatin and gemcitabine chemotherapy for patients with advanced biliary tract cancer (ABC-03): a randomised phase 2 trial.* Lancet Oncol *2015; **16:** 967–78*—In figure 3 of the Appendix of this Article, parts A and B were mislabelled; part A shows overall survival data and part B shows progression-free survival data. The appendix has been corrected as of Aug 31, 2015.

