# Supplementary material for: Cediranib or placebo in combination with cisplatin and gemcitabine chemotherapy for patients with advanced biliary tract cancer (ABC-03): a randomised phase 2 trial
Source: Lancet Oncol. 2015 Aug;16(8):967–78. doi: 10.1016/S1470-2045(15)00139-4 (PMC4648082; doi:10.1016/S1470-2045(15)00139-4)
Supplement: Supplementary appendix [file mmc1.pdf]

THE LANCET Oncology

Supplementary appendix

This appendix formed part of the original submission and has been peer reviewed. We post it as supplied by the authors.

This online publication has been corrected. The corrected version first appeared at thelancet.com/oncology on August 31, 2015.

Supplement to: Valle JW, Wasan H, Lopes A, et al. Cediranib or placebo in combination with cisplatin and gemcitabine chemotherapy for patients with advanced biliary tract cancer (ABC-03): a randomised phase 2 trial. *Lancet Oncol* 2015; published online July 13. http://dx.doi.org/10.1016/S1470-2045(15)00139-4.

a.

**Biomarker**	Categories*	**Forest plot**	**HR** (95% CI)	N	Deaths	**Interaction p-value***
**CA19-9**	<166		0·68 (0·37 - 1·26)	59	43	0·837 **(0.001)**
IU/mL	≥166		0·73 (0·42 - 1·26)	59	54	
**CA125**	<32		0·98 (0·52 - 1·82)	56	40	0·286 (0.103)
IU/mL	≥32		0·62 (0·34 - 1·13)	54	48	
**CEA**	<4.1		1·07 (0·61 - 1·87)	70	50	0·169 (0.237)
μg/L	≥4.1		0·56 (0·32 - 1·00)	53	49	
**Total CK-18**	<721		0·69 (0·38 - 1·25)	60	45	0·357 (0.174)
U/L	≥721		0·97 (0·56 - 1·68)	59	52	
**CTC count**	0		0·70 (0·35 - 1·41)	51	33	0·912 (0.127)
In 7.5ml	≥1		0·65 (0·35 - 1·21)	44	43	
**Ang1**	<1425		1·11 (0·63 - 1·97)	59	48	0·141 (0.746)
pg/ml	≥1425		0·57 (0·32 - 1·02)	58	48	
**Ang2**	<591		0·88 (0·50 - 1·57)	59	47	0·654 (0.295)
pg/ml	≥591		0·72 (0·41 - 1·28)	58	49	
**FGFb**	<167		0·86 (0·48 - 1·55)	59	45	0·960 (0.782)
pg/ml	≥167		0·76 (0·44 - 1·33)	58	51	
**HGF**	<299		0·71 (0·39 - 1·28)	59	45	0·346 (0.658)
pg/ml	≥299		0·95 (0·54 - 1·67)	58	51	
**IL6**	<35		0·82 (0·44 - 1·51)	59	43	0·859 (0.490)
pg/ml	≥35		0·69 (0·40 - 1·21)	58	53	
**IL8**	<44		0·81 (0·45 - 1·47)	59	44	0·716 (0.852)
pg/ml	≥44		0·82 (0·47 - 1·43)	58	52	
**KGF**	<11·6		0·79 (0·44 - 1·44)	59	44	0·986 (0.273)
pg/ml	≥11·6		0·66 (0·37 - 1·19)	58	52	
**PDGFbb**	<162		1·47 (0·83 - 2·61)	59	47	**0·002 (0.006)**
pg/ml	≥162		0·36 (0·20 - 0·67)	58	49	
**PlGF**	<32		0·95 (0·52 - 1·72)	59	44	0·462 (0.292)
pg/ml	≥32		0·63 (0·36 - 1·10)	58	52	
**SDF1b**	<1904		0·63 (0·35 - 1·13)	59	49	0·316 (0.787)
pg/ml	≥1904		0·95 (0·53 - 1·70)	58	47	
**Tie2**	<7462		0·79 (0·43 - 1·44)	59	44	0·918 (0.288)
pg/ml	≥7462		0·79 (0·45 - 1·37)	58	52	
**VEGFA**	<171		1·17 (0·65 - 2·11)	59	44	0·128 (0.471)
pg/ml	≥171		0·52 (0·30 - 0·92)	58	52	
**VEGFC**	<143		0·99 (0·55 - 1·78)	59	46	0·446 (0.154)
pg/ml	≥143		0·65 (0·37 - 1·15)	58	50	
**VEGFR1**	<74		1·00 (0·55 - 1·82)	59	44	0·307 (0.224)
pg/ml	≥74		0·65 (0·37 - 1·13)	58	52	
**VEGFR2**	<6382		0·83 (0·46 - 1·53)	59	44	0·609 (0.618)
pg/ml	≥6382		0·74 (0·43 - 1·28)	58	52	

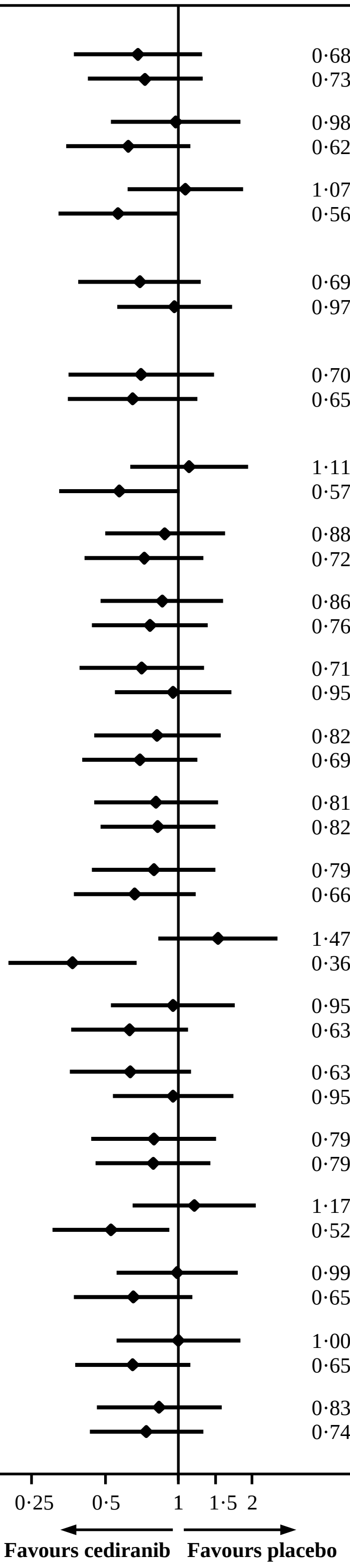

Appendix Figure 3.

Forest plots showing the effect of treatment on OS (**a**) and PFS (**b**) at low and high levels of each baseline biomarker studied. *p-value of interaction with the biomarker as categorical variable. In brackets p-value of the interaction with biomarker as continuous variable.

b.

**Biomarker**	Categories*	**HR** (95% CI)	N	PFS events	**Interaction p-value***
**CA19-9**	<166	0·91 (0·52 - 1·57)	59	53	0·760 (0.161)
IU/mL	≥166	0·73 (0·43 - 1·26)	59	57	
**CA125**	<32	1.07 (0.61 - 1.86)	56	50	0·270 (0.386)
IU/mL	≥32	0.63 (0.35 - 1.11)	54	53	
**CEA**	<4.1	1·06 (0·64 - 1·74)	70	63	0·507 (0.338)
μg/L	≥4.1	0·75 (0·43 - 1·30)	53	52	
**Total CK-18**	<721	0·87 (0·51 - 1·48)	60	56	0·972 (0.823)
U/L	≥721	0·93 (0·55 - 1·58)	59	56	
**CTC count**	0	1·09 (0·61 - 1·96)	51	46	0·172 **(0.002)**
In 7.5ml	≥1	0·64 (0·34 - 1·20)	44	43	
**Ang1**	<1425	1·46 (0·85 - 2·51)	59	56	**0·035** (0.343)
pg/ml	≥1425	0·64 (0·37 - 1·11)	58	54	
**Ang2**	<591	1·29 (0·76 - 2·21)	59	55	0·073 (0.055)
pg/ml	≥591	0·70 (0·41 - 1·20)	58	55	
**FGFb**	<167	1·26 (0·74 - 2·16)	59	54	0·104 (0.320)
pg/ml	≥167	0·70 (0·41 - 1·20)	58	56	
**HGF**	<299	1·07 (0·63 - 1·84)	59	56	0·475 (0.224)
pg/ml	≥299	0·84 (0·49 - 1·43)	58	54	
**IL6**	<35	1·38 (0·80 - 2·36)	59	54	**0·008 (0.030)**
pg/ml	≥35	0·56 (0·32 - 0·96)	58	56	
**IL8**	<44	1·28 (0·75 - 2·21)	59	53	**0·043 (0.018)**
pg/ml	≥44	0·61 (0·36 - 1·05)	58	57	
**KGF**	<11·6	1·21 (0·71 - 2·06)	59	55	0·101 (0.067)
pg/ml	≥11·6	0·66 (0·38 - 1·15)	58	55	
**PDGFbb**	<162	1·52 (0·89 - 2·61)	59	54	**0·004 (0.013)**
pg/ml	≥162	0·47 (0·27 - 0·81)	58	56	
**PlGF**	<32	1·44 (0·83 - 2·48)	59	53	**0·005** (0.147)
pg/ml	≥32	0·52 (0·30 - 0·90)	58	57	
**SDF1b**	<1904	0·76 (0·44 - 1·29)	59	56	0·338 (0.785)
pg/ml	≥1904	1·07 (0·63 - 1·83)	58	54	
**Tie2**	<7462	1·04 (0·61 - 1·79)	59	53	0·504 (0.960)
pg/ml	≥7462	0·83 (0·49 - 1·41)	58	57	
**VEGFA**	<171	1·40 (0·82 - 2·39)	59	55	**0·032** (0.093)
pg/ml	≥171	0·62 (0·36 - 1·07)	58	55	
**VEGFC**	<143	1·23 (0·72 - 2·11)	59	54	0·099 (0.059)
pg/ml	≥143	0·69 (0·40 - 1·17)	58	56	
**VEGFR1**	<74	1·22 (0·71 - 2·10)	59	53	0·103 (0.113)
pg/ml	≥74	0·65 (0·38 - 1·12)	58	57	
**VEGFR2**	<6382	1·16 (0·68 – 1·99)	59	53	0·160 (0.079)
pg/ml	≥6382	0·69 (0·41 - 1·18)	58	57	

Forest plot

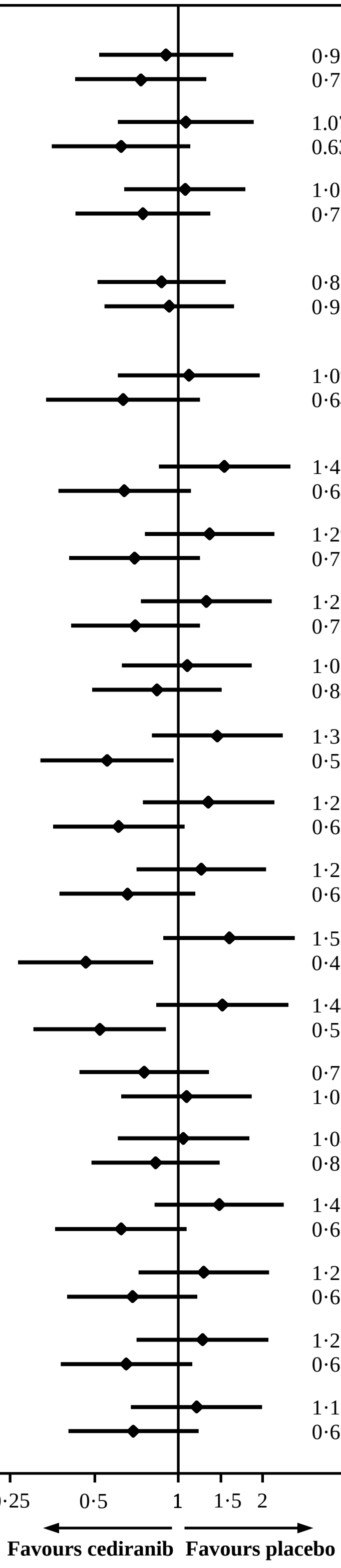

Appendix Figure 3.

Forest plots showing the effect of treatment on OS (**a**) and PFS (**b**) at low and high levels of each baseline biomarker studied. *p-value of interaction with the biomarker as categorical variable. In brackets p-value of the interaction with biomarker as continuous variable.
